# DIGITAL NIST: An examination of the obstacles and opportunities in the digital transformation of NIST’s reference materials

**DOI:** 10.21014/actaimeko.v12i1.1403

**Published:** 2023

**Authors:** William Dinis Camara, Steven Choquette, Katya Delak, Robert Hanisch, Benjamin Long, Melissa Phillips, Jared M. Ragland, Catherine Rimmer

**Affiliations:** 1 National Institute of Standards and Technology (NIST), Gaithersburg, MD 20899, USA

**Keywords:** Digital transformation, digital Reference Materials, DRMC, digital SI, DCC

## Abstract

Early in 2022, NIST embarked on a pilot project to produce digital calibration reports and digital certificates of analysis for reference materials. The goal is to produce examples of digital reports and certificates to assess the scope and challenges of digital transformation in those particular measurement services. This paper focuses on the Reference Material Certificate effort of the pilot project. Our aims for this part of the pilot project are: to generate a digital Reference Material Certificate from certification data; descriptive information about the material, and other data and metadata as needed; to generate a human-readable report from the digital Reference Material Certificate; and to hold a workshop to gather stakeholder feedback. The challenges for NIST include the diverse and complex information presently contained in NIST certificates, converting values to non-SI units to match the needs of stakeholders, and format updates to NIST Reference Material Certificates necessary to allow for machine generation. Other practical challenges include the wide variety of Reference Materials offered by NIST, as well as the needs of internal and external stakeholders. This presentation will report on the progress of the NIST effort and discuss some of the challenges and solutions to producing Digital Reference Material Certificates.

## 1. INTRODUCTION

The digital transformation of metrology is a worldwide movement. For national metrology institutes (NMIs), supporting digital technologies is now an urgent task. NMIs want to support Industry 4.0 and Internet of Things (IoT) concepts, digital twin concepts, and many other digital technologies. Although the activities of NMIs vary, the majority provide measurement services that allow their stakeholders to obtain documented traceability to the International System of Units (SI). Access to the documentation is typically delivered using methods that require a human to review and process the information provided. This may involve extracting pertinent data from paragraphs of text. With the proliferation of digital technologies and communication, a faster and more automated transfer of information is essential for technological growth.

Therefore, the digital delivery of measurement service data is a key and fundamental digital technology that NMIs seek to support. To that end “A universal and flexible structure for digital calibration certificates (DCC)” was developed under the auspices of the European Metrology Program for Innovation and Research (EMPIR) project SmartCom 17IND02 [1]. Practically, the DCC implements an XML schema for producing digital calibration certificates. The DCC continues to be supported and is presently on version 3.1.0; many NMIs are now adapting the DCC for use in their own institutions. However, the DCC in its current version is not suitable for Digital Reference Material Certificates (DRMC) due to the different information, underlying standards and dissemination requirements related to reference materials. A new schema must be created to support the digital delivery of reference materials.

NIST has recognized expertise in many digital technologies, including IoT, cybersecurity, and artificial intelligence (AI), but has not yet attempted to deliver measurement service data in a digital format. In 2022, NIST embarked on a pilot project to produce examples of digital calibration reports and certificates of analysis for NIST Standard Reference Materials (SRMs). The main objective of the pilot project is to assess the resources and effort required to deliver fully digital measurement service data. Like other NMIs, NIST is using the DCC as a starting point. The efforts required to digitize calibration reports and certificates of analysis are separate but related. For the SRM effort, one of the main challenges is that the DCC has been constructed for calibrations, not reference materials. Although many of the elements are similar, there are also significant differences, particularly in the context of metadata. Attempting to put reference material data directly into the DCC as currently constructed, adding additional data fields, and modifying existing data fields will lead to a more difficult-to-use patchwork collection of data fields. Here, we focus on the reference material side of the pilot project, and we note that at the time of writing the pilot project is rapidly developing.

## 2. NIST STANDARD REFERENCE MATERIALS^®^

**Figure 1.**
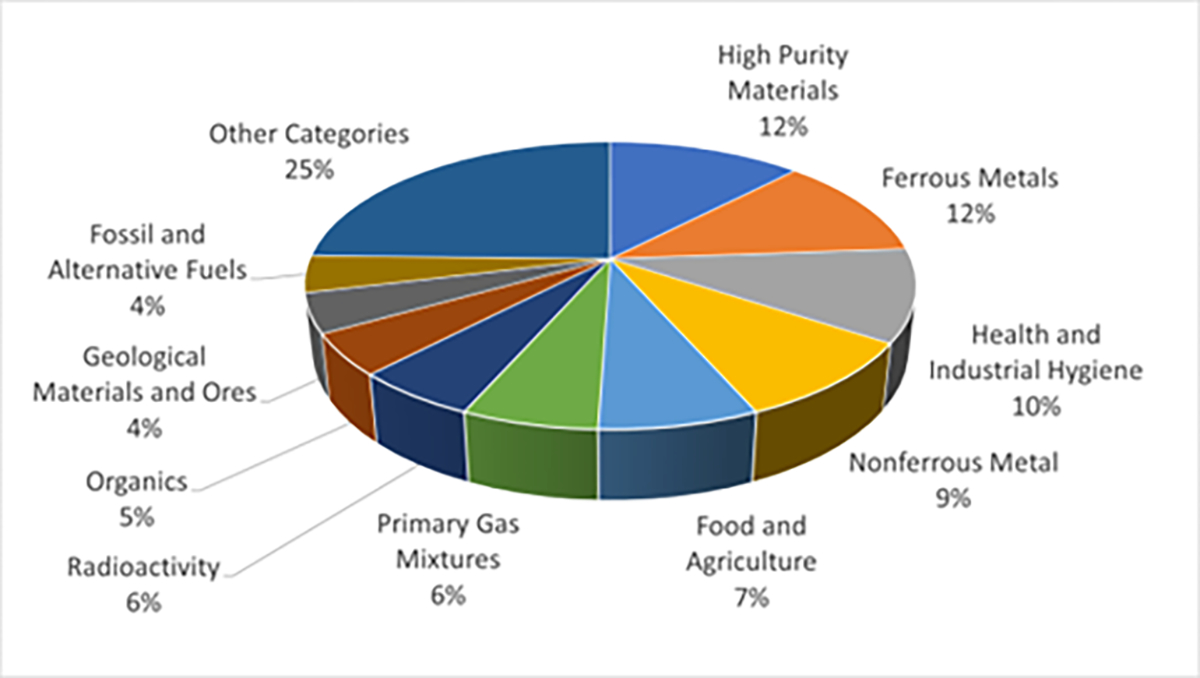
NIST SRMs shown with the percentage of materials from the catalogue in each category.

NIST offers over 1100 Standard Reference Materials in the following categories [2]: Ferrous Metals, Nonferrous Metal, Microanalysis, High Purity Materials, Health and Industrial Hygiene, Inorganics, Primary Gas Mixtures, Fossil and Alternative Fuels, Organics, Food and Agriculture, Geological Materials and Ores, Ceramics and Glasses, Cement, Engine Wear Materials, Forensics, Ion Activity, Polymeric Properties, Thermodynamic Properties, Optical Properties, Radioactivity, Electrical Properties, Metrology, Liquids and Glasses, X-Ray Diffraction, Sizing, Surface Finish, Fire Research, Nanomaterials, and Miscellaneous Performance Engineering Materials. NIST produces a significant number of SRMs in the technical categories covering a wide array of needs. The distribution of the number of SRMs by category is shown in Figure 1.

The large number and diversity of SRMs is a complex challenge for the digital delivery of reference materials and documentation because the types of data and values may vary greatly among the various SRMs. The different technical areas have unique challenges for providing the measurements that stakeholders need.

The quality management system for NIST SRMs is, to the extent allowed by statute and regulation, in conformity with the International Organization for Standardization (ISO) standard 17034. Because the DCC was designed to conform to ISO/IEC 17025, a schema for reference materials requires evaluation of the information required by ISO 17034 and other sources to generate a schema for reference materials before comparison to the DCC to identify similar elements that can be reused between the two.

## 3. CHALLENGES

### 3.1. Digital Certificates

NIST plans to use an internally developed tool, the Configurable Data Curation System (CDCS) [3], to transform SRM metadata and results into digital SRM Certificates based on the new schema being created. The data for the certificates will be stored in a digital repository that will enable easier and higher-quality generation of SRM Certificates.

ISO 17034 and ISO Guide 31 provide general requirements for reference material producers including much of the information that needs to be provided in certificates. In addition, other sources such as NIST policy and customer input provide further information to be contained in certificates. Since the DCC is designed around a different standard, ISO/IEC 17025, that requires different information, it is not a suitable schema for digital reference materials. A new schema must be researched and created based on the requirements for reference material certificates with input from NIST stakeholders and in collaboration with other national metrology institutes. Since similarities will exist between the new schema and the DCC, they will need to be compared to determine where similarities may be combined for more efficient digital delivery of measurement services information.

The materials used for NIST SRMs cover a wide range of technical areas. Due to the varying types of data in these materials, NIST supports multiple approaches to assigning values to best fit the homogeneity of each material. Materials with between-unit homogeneity have certificates that can apply to multiple units across a batch or lot of material. Most of the certificates for these NIST SRMs are publicly available. However, materials that have only within-unit homogeneity require a distinct certificate for each unit. For these serialized materials, the certificate with data values is generally available only to the customer who purchased the unit and will necessitate limitations and additional security measures on how each certificate is delivered and accessed.

**Figure 2.**
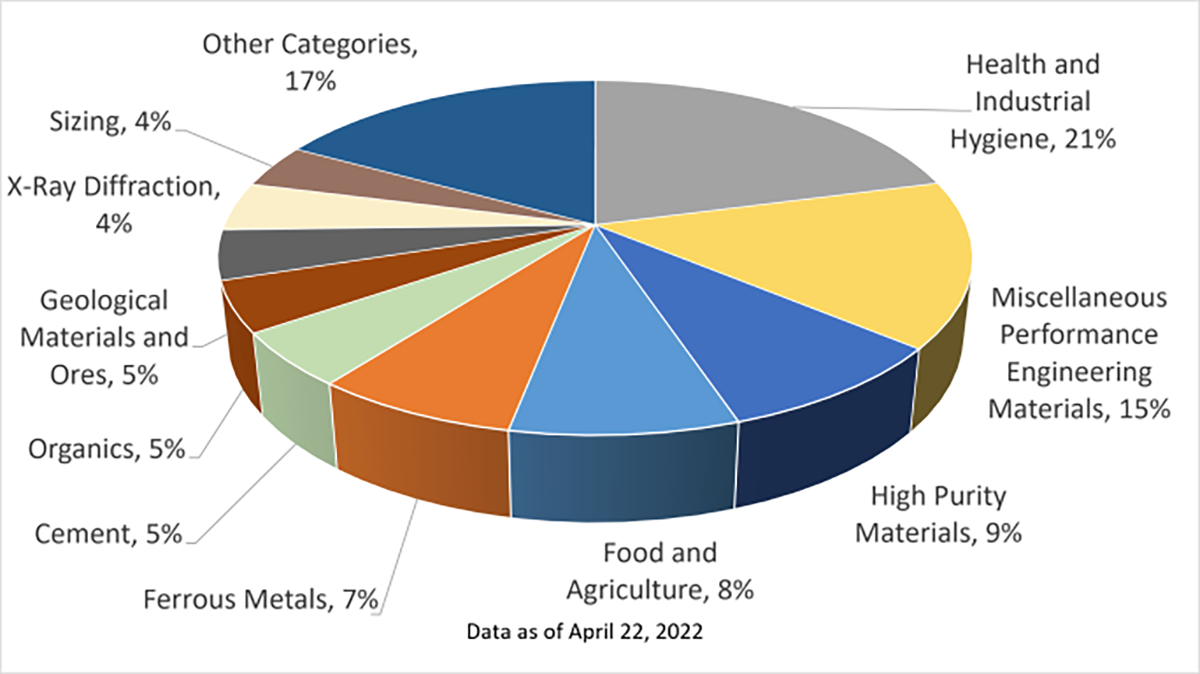
NIST SRMs shown with the percentage of sales of materials in each category.

Additionally, values may be reported in multiple ways in certificates, e.g., a single value with an uncertainty, multiple values and uncertainties, and DNA sequences. NIST favours flexibility in delivering values in the units preferred by our stakeholders since our user communities use different units including both SI and non-SI units in their daily measurements. For example, users measuring for ignition resistance testing may use values expressed as a percentage, while users measuring coating thickness may use measurements to be expressed in mils. Providing flexibility to the SRM stakeholders is fundamental to creating a digital reference material certificate that will satisfy customer needs in disparate technical areas. The sale of NIST SRMs is not concentrated in any particular area of material and the needs of each of these areas must be regarded. The distribution of SRM sales by category is shown in Figure 2.

Another challenge present in certificates is the variety of special characters that must be accommodated. Greek letters, superscripts, subscripts and other symbols are frequently included and must be accommodated in any digital solution.

Additionally, reference material certificates are not static and may change over time. A certificate for a reference material may be updated to include new values that have been measured, for changes in expiration dates, or for numerous other reasons. While each version of a certificate contains unique information, the connection to historical versions of the documents needs to be maintained. The users of reference materials must ensure that they are using the current version of the certificate for their material. Each year NIST updates between 150 and 200 certificates.

To populate a digital certificate, information must be collated from multiple sources. Much of the metadata used to populate the digital certificates is stored electronically in databases. Other information is manually curated and added. New code must be written to extract the electronically stored information for inclusion in certificates. For manually added information, new methods of storing the data must be created to automate inclusion.

### 3.2. Human-Readable Certificates

**Figure 3.**
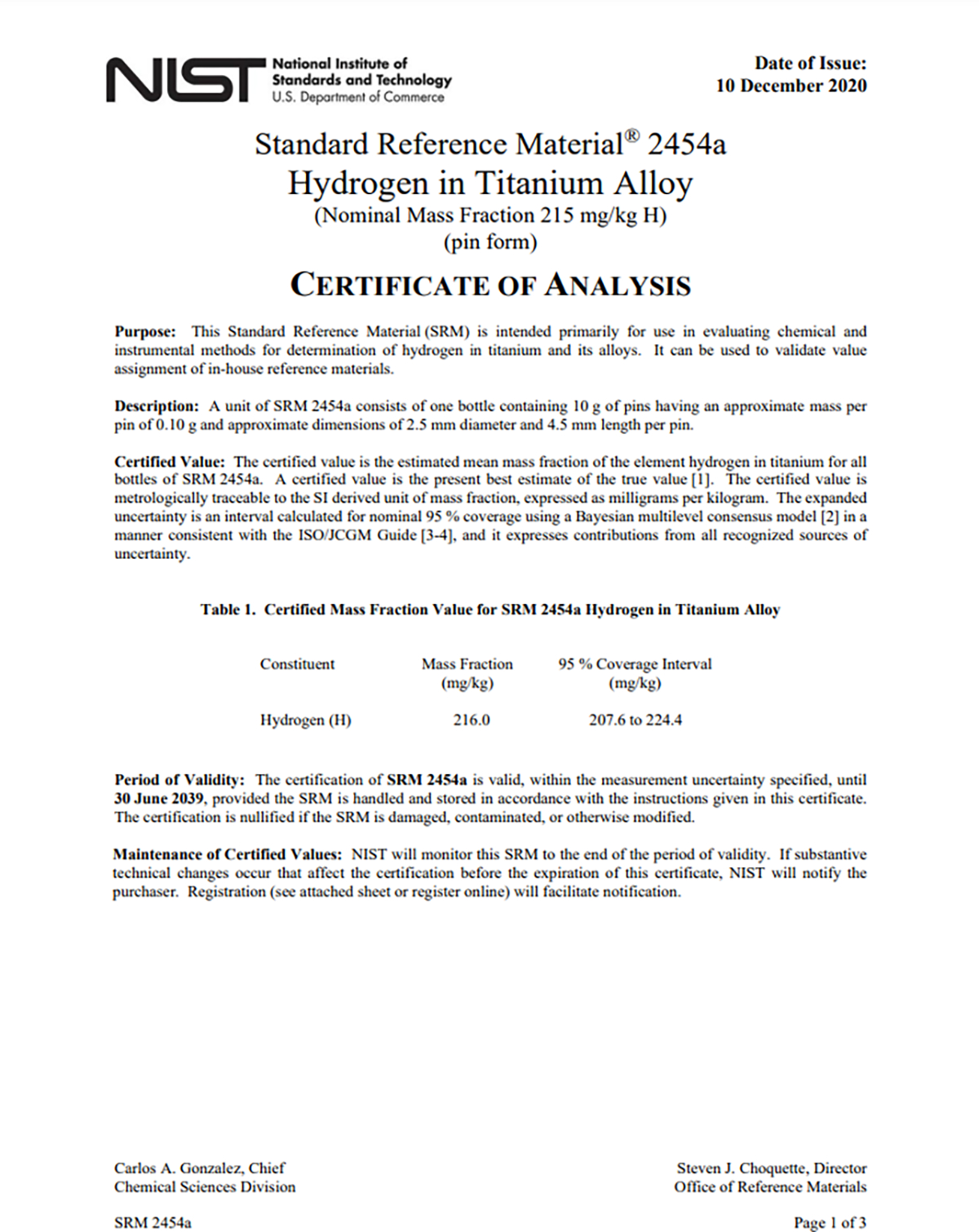
The first page of the certificate for SRM 2454a Hydrogen in Titanium Alloy (Nominal Mass Fraction 215 mg/kg H) (pin form).

The creation of digital reference material certificates to enable machine-to-machine communication is essential to the future of metrology. However human-readable versions of the certificates will be necessary for at least the foreseeable future. An example of the first page of a current human-readable certificate is shown in Figure 3. Users of NIST SRMs will utilize the human-readable versions for information about the material including storage, usage instructions, and other required product information. These versions also provide information about the materials to the stakeholders that aid in determining whether the SRM is the best fit for their needs. These human-readable versions of digital reference material certificates must be formatted such that stakeholders can easily extract the information they need for their purposes. Some of the elements that currently exist in NIST certificates of analysis are intended primarily for the human reader and should continue to be included in documentation provided to the customers.

The certificates for NIST SRMs have changed over the more than 100 years that NIST has been producing them. Early certificates contained only values and uncertainties whereas modern certificates include more information about the source and preparation of the materials, more detailed information on storage, instructions for use, and other information required to educate customers and inform them of measurement concerns when using the materials. The schema for a DRMC must take into account the wide range of information that has been included in the certificates for the benefit of the customers. Many NIST reference material certificates include figures, photos, and plots of results that communicate pertinent information or help the user to visualize tabular data.

The need for these types of items to be machine-readable must be investigated. However, they must be incorporated into the DRMC schema so that they can be included in the human-readable certificate. The full benefit of a digital reference material certificate cannot be realized if human editing of a certificate after its generation is required.

### 3.3. Tools for Finding the Appropriate SRM

The creation of a full digital repository of information will have the added benefit of improving searching for SRMs. Currently customers utilize manual tools that can search a limited set of information in the certificate to compare multiple SRMs available from NIST. Customers access individual certificates and compare information about materials that do not share the same measurands, measurement units, or matrices. This approach does not accommodate complex searches and consumes significant time and resources from the customer.

The digital repository will enable new, more automated tools to be created, perhaps even by private companies, that will allow for quicker comparison of available SRMs to identify the one that best suits the need of the customer. A first step may be to provide searching tools that are usable through a web interface. Additional tools that would allow two machines to directly interact may be possible in the future. Perhaps there will be a day when an instrument in the laboratory searches the NIST SRM catalogue and informs the user of the appropriate material to acquire.

One of the greatest challenges to successful automated searches is the ability to compare measurements that use different units. Due to the volume of possible conversions, they cannot be calculated ahead of time and stored. Rather the schema must allow for these types of calculations to be done in real time as the search is conducted.

## 4. NIST WORKSHOP

NIST hosted an international workshop on 28–29 September 2022, for stakeholders of its calibration services and reference materials. The workshop introduced stakeholders to the results of the pilot study, specifically beta versions of digital calibration reports and certificates of analysis and their corresponding human-readable versions and seek stakeholder feedback on these beta versions. The workshop was an opportunity for NIST to learn how stakeholders are responding to the changes towards more digital implementations of metrology, any solutions that they are implementing, and to better understand customer needs in this area.

Participants for the workshop were identified through a questionnaire that was sent to customers of NIST measurement services through email and was posted on the NIST website. The questionnaire gauged stakeholder interest in participating in the workshop and topics for digital transformation, such as digital calibration reports, certificates of analysis, digital traceability and security, and middleware for digital reports and certificates of analysis. NIST will report on the results of the workshop in a separate document.

Following the workshop, NIST is developing a strategy for full digital transformation of its measurement services.

## 5. FUTURE DIRECTIONS

The current version of the DCC is not adequate for use in creating a DRMC. Continued development of a new model to conform to the specifications of Reference Materials is needed to have a model capable of handling the metadata associated with these materials.

There are clear benefits ranging from within organization to worldwide for the digital transformation of reference materials and other measurement services. Coordination and development of a model for reference materials that can be used worldwide will be essential to a successful transformation. NIST will continue to monitor the DX being developed in the next version of the DCC. For Reference Materials the international community is watching the direction that national metrology institutes take in leading this effort. The work currently ongoing will provide the foundation for the transition to digital for Reference Materials.
